# Valorization of Fine Recycled Aggregates Contaminated with Gypsum Residues: Characterization and Evaluation of the Risk for Secondary Ettringite Formation

**DOI:** 10.3390/ma13214866

**Published:** 2020-10-30

**Authors:** Charlotte Colman, David Bulteel, Sébastien Rémond, Zengfeng Zhao, Luc Courard

**Affiliations:** 1 Laboratoire de Génie Civil et géo-Environnement, Université de Lille, IMT Lille Douai, ULR 4515-LGCgE, F-59000 Lille, France; david.bulteel@imt-lille-douai.fr; 2 Urban and Environmental Engineering, GeMMe Building Materials, University of Liège, 4000 Liège, Belgium; zengfeng.zhao@uliege.be (Z.Z.); luc.courard@uliege.be (L.C.); 3 Laboratoire de Mécanique Gabriel Lamé (LaMé), Université d’Orléans, 45100 Orléans, France; sebastien.remond@univ-orleans.fr

**Keywords:** fine recycled aggregates, sulfate attack, construction and demolition waste, secondary ettringite formation

## Abstract

Fine recycled aggregates (FRA) (0/4 mm) are up to now not valorized on a high enough level because of characteristics like an elevated water absorption, higher fines content, and the presence of contaminations. Leftover gypsum residues from the construction site can cause internal sulfate attack when FRA are incorporated into new structures. Concern about this deteriorating reaction plays an important role in the rejection of FRA. In this study, samples of FRA from different recycling centers were characterized and incorporated into mortars. They were then subjected to swelling tests in order to evaluate the development of sulfate attack. Reference materials with different amounts of sulfates were used as a comparison. Results showed a variable sulfate content in industrial FRA, depending heavily on the source of the materials. In all but one case, the total amounts surpassed the acceptable sulfate contents specified in the European standard EN 206, meaning the FRA would be rejected for reuse in concrete. Nevertheless, swelling tests demonstrated that these contamination levels did not pose a risk for sulfate attack. These results indicated that the incorporation of FRA leads to acceptable mechanical performances and that the sulfate limit could be reviewed to be less strict.

## 1. Introduction

The building sector is infamously known as the 40%-industry, using 40% of global energy and resources, and is responsible for one-third of our greenhouse gas emissions [1]. Nowadays, 1 m^3^ of concrete, corresponding to more or less 2 tonnes, is being produced per person per year. The climate impact of the construction industry can be reduced by 77% by core material separation and its recycling or reuse [2]. Accounting for approximately 25–30% of all waste generated, Construction and Demolition Waste (C&DW) is one of the heaviest and most voluminous waste streams in the EU and as such has been identified as a priority waste stream by the European Union [3]. C&DW consists of numerous materials, including concrete, bricks, gypsum, wood, glass, metals, plastic, and excavated soil [4]. The major fraction of C&DW however is mineral waste, which has a high potential for recycling and reuse [5]. One of the objectives posed in the Waste Framework Directive (WFD) of the European Union (2008/98/EC) is to reuse a minimum of 70% of C&DW by 2020 [6], either by backfilling as road bases or—the most preferrable high-quality application—reusing them in the form of Recycled Aggregates (RA). RA are reprocessed from C&DW and can be used in mortar or concrete as a replacement for natural aggregates (NA) [7,8]. This practice reduces the amount of debris disposed of in landfills, reduces the rate of natural resource depletion, and provides energy, cost and transport savings [9]; 1.7 tonnes of these recycled aggregates are produced per person per year in Europe, waiting to be valorized [10].

The use of coarse recycled aggregates has been shown to produce concrete with acceptable properties [11,12,13,14,15]. Fine recycled aggregates (FRA), however, have more nefarious characteristics and their incorporation into a new concrete is up to now generally avoided [16,17]. These properties include—among others—a higher water absorption [18,19]; a lower density; and the presence of contaminations from the construction or demolition site such as plaster, bricks, wood, etc. [20,21]. Existing studies often focus on mechanical properties, and research is needed on the durability aspects of the incorporation of FRA.

Gypsum (CaSO_4_·2H_2_O) is used in the construction sector firstly as an addition to Portland cement, to regulate the setting time of cement and prevent a flash set [22]. Besides that, gypsum is the major constituent of plaster walls in buildings. Demolished concrete particles contain adherent cement and plaster, which will lead to a certain sulfate content in recycled aggregates after the crushing process. The effective sulfate concentration in RA will of course depend on the type of the source concrete but most stocks of RA in recycling centers are mixed from different sources and demolition sites: a sulfate contamination is a very realistic concern for RA. Larger concrete and gypsum particles can be separated from each other based on a difference in color [23] or density [24], but these techniques are not applicable on the smallest size fractions. Especially in FRA, gypsum is an important contaminant to be considered: the water-soluble sulfates coming from the gypsum particles strongly limit their valorization potential [25].

Sulfate attack is a deteriorating process where sulfates dissolve in water and react with aluminate hydrates in a hardened cement paste to form secondary ettringite. It is assumed that this mineral exerts a pressure on its surrounding cement paste and thereby causes a volumetric deformation [26]. Macroscopically, the concrete structure will show swelling behavior and the formation of (micro)cracks. Primary ettringite is a normal hydration product in the cement paste: it is only secondary ettringite, formed in an already rigid cement matrix, that risks causing a swelling reaction.

A distinction can be made between different types of sulfate attack. External sulfate attack happens when the sulfates diffuse into the concrete from an aggressive environment [27]. Another reaction called Delayed Ettringite Formation (DEF) occurs when primary ettringite is destroyed by high curing temperatures and formed anew in a hardened cement paste. The sulfates in this case come from cement, an internal source [22,26,28] .

While external sulfate attack and Delayed Ettringite Formation are well researched and understood, the reaction caused by the presence of gypsum in FRA is not. The gypsum residues contaminating FRA are another internal source of sulfates and unlike with the DEF reaction, high curing temperatures are not needed to observe the swelling effect of ettringite formation. The term “secondary ettringite formation” will be used to distinguish this reaction from DEF.

To keep the risk for secondary ettringite formation at a reasonable level, the current water soluble sulfate limit in coarse recycled aggregates is established at 0.2% by EN 206 [29], with no specific mention of FRA. The conclusions of recent durability studies indicate a higher level should be made possible [30], specifically up to contents of 0.3% [31].

In this study, different sources of FRA were characterized for their sulfate content, water absorption, and size distribution; this provided information about the variability in characteristics between industrially available FRA. The materials were then incorporated into mortars. Swelling tests performed on these mortars indicated whether or not the found contamination levels did indeed cause a deterioration. Ultimately, these results might provide a better understanding of the long term effects of sulfate attack in the context of construction and demolition waste and will promote the use of these recycled materials in the building sector.

## 2. Materials and Methods

Six samples of recycled aggregates were collected from recycling centres in Belgium, of which only the 0/4 mm fraction was characterized and used. They are named “A” to “F”. In total, 3 recycling plants were sampled: of the six FRA samples, four were from different batches of the same recycling plant. As a reference sample, a pure FRA was also made in the laboratory by fabricating and crushing a standard concrete. This sample is called “REF”. The composition of this original concrete was designed to obtain a consistency class S3 and strength class C30/37, and was made with CEM I and limestone aggregates. After 90 days of curing, this concrete was crushed by a jaw crusher and the resulting 0/4 mm fraction was used as FRA. The use of this “model” FRA as a reference gave exact control of the chemical composition of the materials and removed any possible variability or contamination at the level of the aggregates by chlorides, organics, etc. The results on FRA are compared with those from a natural limestone aggregate called ’NS’.

### 2.1. Characterization

All FRA samples were characterized for their size distribution according to EN 933-1 [32].

Only water-soluble sulfates contribute to secondary ettringite formation. The procedure described in EN 1744-1 [33] for the determination of water-soluble sulfates in recycled aggregates was followed with two adaptations to the testing protocol. First, an elevated temperature to extract the sulfates was not used because gypsum exhibits a retrograde solubility [34]. Second, sulfate concentrations were measured with ion chromatography instead of spectrophotometry, which is an easier, safer, and more precise analytical technique [35].

Water absorption and particle density of the FRA were determined via the method described by Zhao et al. [18]. Characterization techniques for natural aggregates, described in EN 1097-6 [36], consistently underestimate the water absorption of FRA because of the fineness and agglomeration issues between the particles. The method, designed in response to this difficulty, by IFSTTAR [37] seems to overestimate the water absorption of FRA, but works well for particles in the 0.5/4 mm range. Thanks to an excellent correlation between the hardened cement paste content or mass loss at 475 ${}^{\circ }$C and the water absorption, the water absorption of the fines can then be extrapolated. Using the water absorption of each size fraction (either measured for the coarser particles or calculated for the fines) is more accurate than using either of the two mentioned experimental methods for the whole 0/4 mm bulk [38].

### 2.2. Swelling Tests

The industrial FRA were used in the manufacture of mortars, to monitor their swelling over the course of 6 months. Three extra mixes were prepared to serve as reference samples:

- Two mixes that will compare the industrial FRA with either a natural aggregate (named “NS”) and a pure crushed concrete (the reference FRA named “REF”). Both the natural and the recycled aggregate are manually contaminated with 0.5 mass% of gypsum. This 0.5% of gypsum corresponds to to 0.29% of sulfates, which reflects the sulfate contents found in industrial FRA. In the case of FRA, this manual contamination is in addition to the water soluble sulfates already found during the characterization, bringing its total sulfate content to 0.47%.
- One mix made with the reference FRA and a very high gypsum content of 5 mass%— corresponding to 2.9% of sulfates—to exaggerate the consequences of sulfate attack. Again, this manual contamination is in addition to the sulfates already present in this FRA, bringing the total sulfate content to 3.08%.

The gypsum used to contaminate the aggregates was a CaSO_4_·2H_2_O powder (D50 13 μm) obtained from VWR Chemicals.

In order to compare only the influence of the different sulfate contents of the sands, other variations between the different aggregate types were reduced as much as possible:

- To account for their difference in size distribution, all aggregates were recomposed to match the size distribution of the reference FRA. This adaptation caused a slight change in the total sulfate content, water absorption, and density. These new values were recalculated.
- To account for their difference in density, a volumetric equivalent of every aggregate was added to the mortars instead of a mass equivalent, to keep the aggregate envelope volume constant.
- To account for their difference in water absorption, all aggregates were pre-saturated one week before mixing, with their absorbed water and 10% of the mixing water. This assures the same amount of effective water in all mixes, proven to be an important factor in the swelling process [39].

The standard procedure described in EN 196-1 [40] for mortar fabrication was followed. A CEM 52.5 N from HOLCIM in Belgium was used for all mortars.

To follow the development of the internal sulfate attack reaction, the mortar specimens were subjected to different tests. The mass, length [41], and resonance frequency were recorded weekly to observe features of sulfate attack such as swelling and possible internal cracking. Length measurements were performed with a digital length comparator, which gives the length of a mortar bar accurately up to 0.001 mm, relative to an Invar reference rod. At 7, 28, 90, and 180 days the mortars were characterized mechanically for their flexural and compressive strength [40]. One sample that showed significant swelling was analyzed with XRD using a Bruker D8 Advance diffractometer according to the powder diffraction method with a Co Kα_1_ radiation, sweep from 10${}^{\circ }$ to 200${}^{\circ }$ 2θ. Every described test was done for 3 replicate mortars.

## 3. Results and Discussion

### 3.1. Characterization

Of the six collected FRA samples, four were from different batches of the same recycling plant. Because of their small particle sizes, it was not possible to obtain a detailed composition of these materials. The industrial sources named these samples “mixed aggregates”, indicating that next to concrete also bricks and other construction materials can be present. Their original characteristics are shown in Table 1. To obtain FRA samples ready for characterization and subsequent incorporation into mortars, they were dried at 40 ${}^{\circ }$C and sieved to keep the 0/4 mm fraction. From the pictures in Table 1, a large variability between the different sources can be seen, and contaminations with soil, brick, wood, plastic, or gypsum particles. Their impurity becomes especially clear when compared to the pure crushed concrete “REF”.

**Figure 1:**
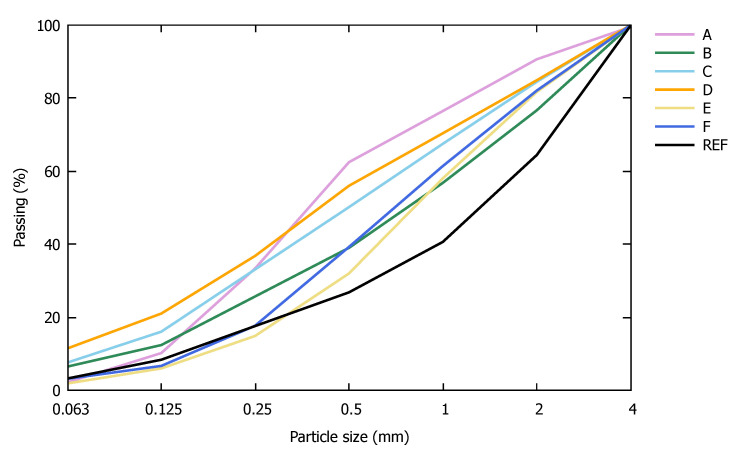
Particle size distribution of the used fine recycled aggregates (FRA).

The particle size distribution of the FRA samples is shown in Figure 1. A very high variability in characteristics was found between the different industrial sources.

**Figure 2:**
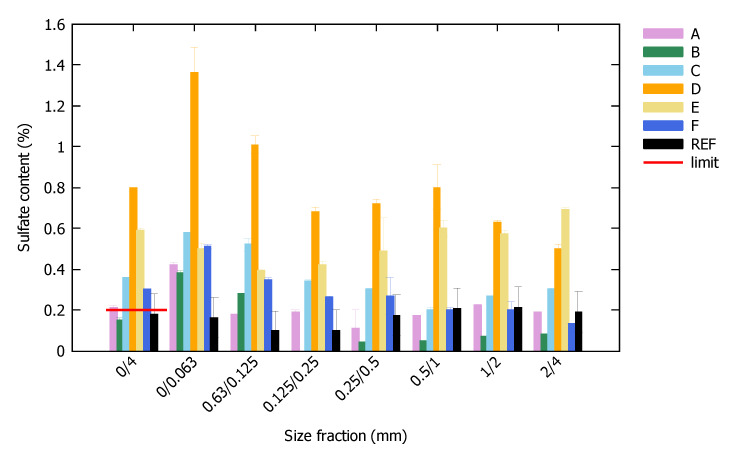
Water soluble sulfate content of the FRA in total and per size fraction.

In Figure 2, the water-soluble sulfate content of the FRA is shown for the total 0/4 mm sample as well as per size fraction. All but one source of FRA surpassed the maximum allowable sulfate limit of 0.2% specified by EN 206 [29], indicating they would be rejected for use in a new concrete. The reference FRA, which was a pure uncontaminated crushed concrete, also contained 0.18% of sulfates, which originated from the leaching of cement particles.

Sulfates are predominantly present in the smaller size fractions, except for sample E and REF. This is explained by the brittleness of gypsum during the handling process of construction and demolition waste, and by the increasing presence of cement particles in the finer size fractions [42,43,44]. The sulfates accumulating to the finer fractions partially explains why—contrary to coarse recycled aggregates—FRA are not valorized.

Table 2 summarizes other characterization results. Again, a large difference in characteristics was noticed, proving the necessity of a thorough characterization of FRA before incorporating them into a mix. An elevated water absorption is one of the key aspects of recycled aggregates, and these ranged from 6.1% to 14.6% over the different samples. No apparent correlation was found between this water absorption and the sulfate content (Figure 2) or the amount of fine particles (Figure 1).

### 3.2. Swelling Tests

Recomposing all aggregates to obtain a uniform size distribution slightly changed their total water absorption, density, and sulfate content. The recalculated values of these recomposed aggregates are shown in Table 2. Taking these values into account, the composition of the mortars was calculated and shown in Table 3. An amount of added gypsum was then recalculated into the corresponding amount of sulfates, giving the total water soluble sulfate content for each mortar in the sample name. These mixes were made in triplicate.

**Figure 3:**
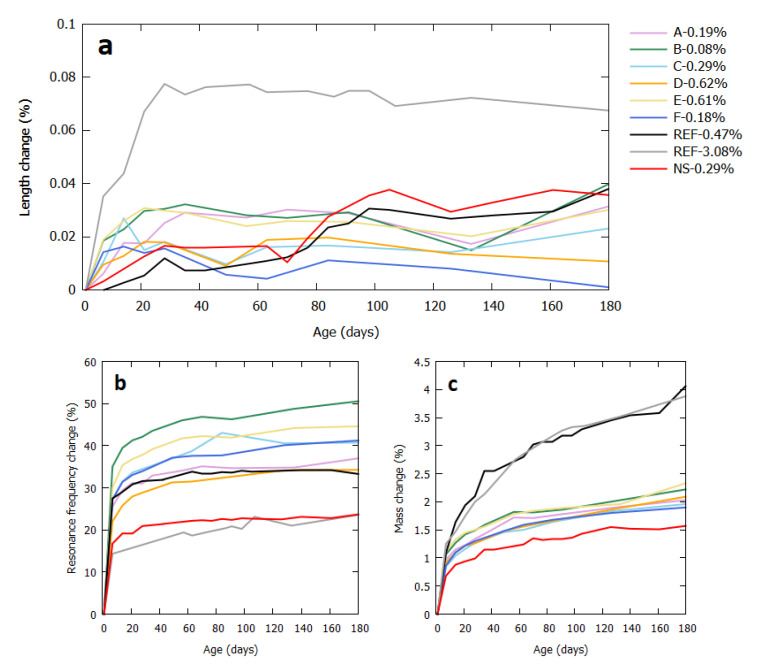
Six-month swelling behavior of the mortar samples, including their length change (**a**), resonance frequency change (**b**), and mass change (**c**).

The 6-month swelling behavior of these mortars is shown in Figure 3. Except for “F-0.18%” which showed a lower expansion, no statistical differences (*p* < 0.05) were found between the length changes of the industrial FRA sources and the 2 reference mortars with similar sulfate contents. More information about statistical differences between the results can be found in Table S1 and Figure S1 in the Supplementary Information. There was also no correlation between the absolute swelling amount and the sulfate content of these mortar mixes. It was, however, very clear that sulfates do indeed cause a swelling reaction—as can be seen from the curve of REF-3.08%, but an exaggerated contamination level was needed for this result. The sulfates present in the industrial FRA were not enough to provoke a significant swelling reaction even though they largely surpassed the 0.2% limit. The length change curve of sample REF-3.08% stabilized after one month, which is a much faster reaction than seen in literature for other types of sulfate deteriorations like DEF or external sulfate attack, that often go on over multiple years [28,45,46,47].

The resonance frequency test is a measure for the internal damage in the mortar sample. If microcracks were formed, they would manifest as irregularities in their frequency curves, but this was not the case for these mortars. Even though significant swelling was attained for REF-3.08%, it was nevertheless not severe enough to cause internal fissuration.

The mass of the mortars kept steadily increasing each week, because of ingress of the water in which they were kept. The mortars made with the reference FRA gained more mass, because of their irregular shape which captures more air into the mixture than a round aggregate [30,48]. For the reference FRA specifically, a high air content in mixes with this material was already observed [49]. Industrially fabricated FRA are rounder by nature than laboratory crushed FRA [50], explaining why their results lie between those of the natural aggregates and the reference FRA.

**Figure 4:**
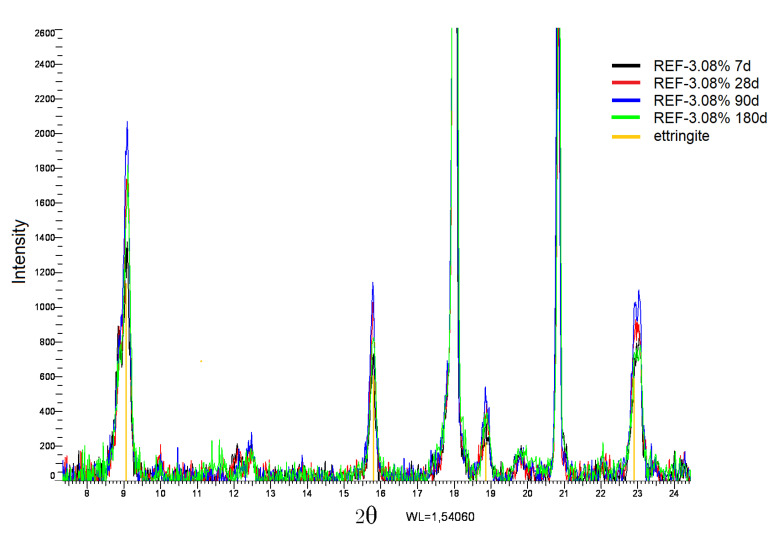
XRD analysis of “REF-3.08%” at 7, 28, 90, and 180 days.

An XRD analysis on “REF-3.08%” (Figure 4) at different moments shows how ettringite peaks become more intense with age, corresponding well to the observed swelling results. The biggest growth for the ettringite peaks is between 7, 28, and 90 days, after which the intensity stabilizes and even decreases slightly at 180 days.

**Figure 5:**
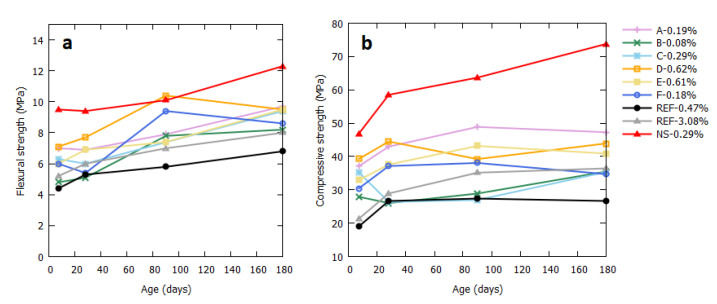
Flexural (**a**) and compressive (**b**) strength of the mortar samples after 7, 28, 90, and 180 days.

Figure 5 shows the flexural and compressive strength of the mortar samples after 7, 28, 90, and 180 days. As expected, the mortar with natural aggregates has a better mechanical performance than those with the reference FRA. The heavily contaminated sample REF-3.08% showed a higher flexural and compressive strength than REF-0.47%, even though it underwent significant swelling. The samples with the industrial FRA performed between the natural aggregates and the reference FRA, confirming their higher quality compared to a pure crushed concrete. This could again be due to the irregular shape of the REF aggregates: the higher air content in these mixes affected their mechanical resistance.

## 4. Conclusions

Between the three different sampled recycling centers and even between different samples from the same center, a large variability was found in the FRA they offered. Results showed their water absorptions ranging from 6.1% to 14.6% and sulfate contents from 0.15% to 0.80%. Both of these characteristics increased with particle fineness, explaining how small particles sizes can be more difficult to incorporate than coarser grains.

Pure, uncontaminated, crushed concrete had a sulfate content of 0.18%, showing that sulfates are not only originating from gypsum but also residual cement particles. Regardless of their source, all water soluble sulfates can contribute to sulfate attack. Mortars made with this reference recycled material showed a lower compressive strength than those with industrial FRA. Lots of research done on the incorporation of FRA in mortars or concrete is done with reference recycled materials, so this could mean that the quality of industrial FRA is underestimated.

Swelling tests on mortars showed that no harmful swelling occurred when industrial FRA were incorporated. While internal sulfate attack caused by contaminated FRA is definitely a relevant deteriorating reaction, exaggerated sulfate amounts were necessary to provoke any swelling. The sulfate contents found in industrial FRA, while still largely surpassing the 0.2% limit posed in EN 206, did not pose this threat to the mechanical stability of the mortar.

For these reasons, FRA from recycling centers should be considered as a viable material to incorporate in mortars, provided that the mix design is adapted to account for their lower density and higher water absorption. Future work should focus on upscaling these tests to concrete, to further evaluate the established sulfate limit.

Based on these results, the sulfate limit of 0.2% posed in EN 206 could be reconsidered to be less strict. Mortars made with sulfate contents of up to 0.6% did not show any significant deteriorating reaction.

## Figures and Tables

**Table 1:** The different samples of aggregates and their original size distribution. Pictures shown are after drying at 40 ${}^{\circ }$C and separating of the 0/4 mm fraction. The “model” FRA and natural aggegrate that were used for reference mortars are also presented.

A	B	C	D
0/4	0/10	0/32	0/20
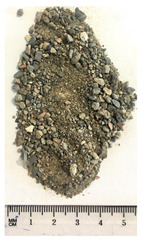	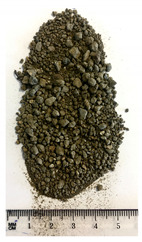	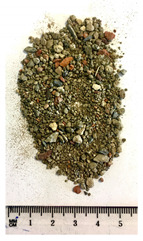	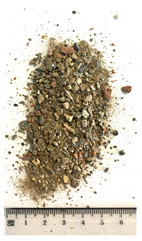
E	F	FRA	NS
0/90	0/10		
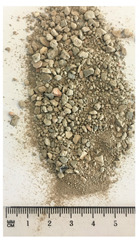	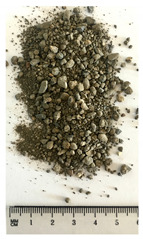	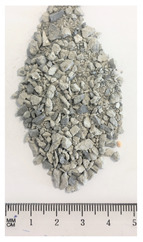	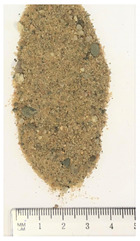

**Table 2:** Characterization of the used FRA before recomposition and effective values in the mortars after recomposition.

	Water Absorption		SSD Particle Density		SO_4_^2^${}^{-}$ Content	
	(%)		(g/cm^3^)		(%)	
**Source**	**Original**	**Recomposed**	**Original**	**Recomposed**	**Original**	**Recomposed**
A	6.1	7.1	2.14	2.15	0.21	0.19
B	8.9	9.7	2.10	2.00	0.15	0.08
C	11.5	10.6	1.99	1.97	0.36	0.29
D	10.6	8.8	2.00	1.92	0.80	0.62
E	14.6	12.6	1.97	1.92	0.59	0.61
F	13.0	11.8	1.96	1.85	0.30	0.18
REF	9.8		1.95		0.18	

**Table 3:** Compositions, in g, of the mortars, using the values of the recomposed FRA from Table 2. The samples are named after the source of their FRA, and the water soluble sulfate content in their mix expressed as a mass % of the aggregate fraction.

Name	Cement	Water		Aggregate	Extra Gypsum
		Effective	Absorbed	(0/4 mm)	
A-0.19%	1350	675	79.15	1116.3	0
B-0.08%	1350	675	100.32	1038.5	0
C-0.29%	1350	675	108.22	1022.9	0
D-0.62%	1350	675	87.33	996.9	0
E-0.61%	1350	675	125.71	996.9	0
F-0.18%	1350	675	113.16	960.6	0
REF-0.47%	1350	675	98.52	1007.4	5.1
REF-3.08%	1350	675	94.07	961.9	50.6
NS-0.29%	1350	675	0	1343.2	6.8

## Supplementary Materials

The following are available online at https://www.mdpi.com/1996-1944/13/21/4866/s1, Figure S1: Swelling curve corresponding to Figure 3a, shown with error bars, Table S1: T-test results of the swelling curves. A red color means that samples differed significantly from each other for *p* < 0.05.
