# Supplementary material for: Valorization of Fine Recycled Aggregates Contaminated with Gypsum Residues: Characterization and Evaluation of the Risk for Secondary Ettringite Formation
Source: Materials (Basel). 2020 Oct 30;13(21):4866. doi: 10.3390/ma13214866 (PMC7662506; doi:10.3390/ma13214866)
Supplement: Supplementary file 1 [file materials-13-04866-s001.pdf]

materials

Supplementary Information

Valorization of Fine Recycled Aggregates Contaminated with Gypsum Residues: Characterization and Evaluation of the Risk for Secondary Ettringite Formation

Charlotte Colman [1,2,*], David Bulteel [1], Sébastien Rémond [3], Zengfeng Zhao [2] and Luc Courard [2]

[1] Laboratoire de Génie Civil et géo-Environnement, Université de Lille, IMT Lille Douai, ULR 4515 - LGCgE, F-59000 Lille, France; david.bulteel@imt-lille-douai.fr
[2] Urban and Environmental Engineering, GeMMe Building Materials, University of Liège, 4000 Liège, Belgium; zengfeng.zhao@uliege.be (Z.Z.); luc.courard@uliege.be (L.C.)
[3] Université d'Orléans, Université de Tours, INSA CVL, EA 7494 LaMé, France; sebastien.remond@univ-orleans.fr
* Correspondence: charlotte.colman@uliege.be

Received: 2 October 2020; Accepted: 26 October 2020; Published: date

Table S1. T-test results of the swelling curves. A red color means that samples differed significantly from each other for $p < 0.05$.

	A-0.19%	B-0.08%	C-0.29%	D-0.62%	E-0.61%	F-0.18%	REF-0.47%	REF-3.08%	NS-0.29%
A-0.19%									
B-0.08%									
C-0.29%									
D-0.62%									
E-0.61%									
F-0.18%									
REF-0.47%									
REF-3.08%									
NS-0.29%									

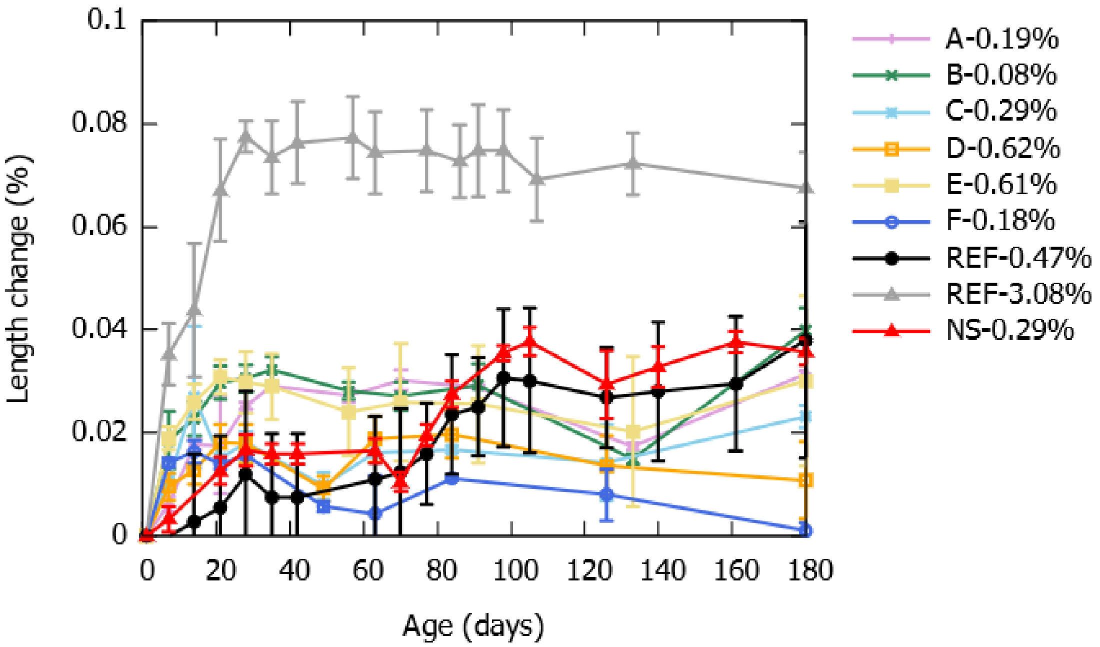

Figure S1. Swelling curve corresponding to Figure 3a, shown with error bars.

Publisher's Note: MDPI stays neutral with regard to jurisdictional claims in published maps and institutional affiliations.

© 2020 by the authors. Submitted for possible open access publication under the terms and conditions of the Creative Commons Attribution (CC BY) license (http://creativecommons.org/licenses/by/4.0/).
